# Sexual Behavior Pattern and Related Factors in Women with Breast Cancer in Iran

**DOI:** 10.5539/gjhs.v8n7p266

**Published:** 2015-12-16

**Authors:** Sanaz Rashidi, Forouzandeh Dashti

**Affiliations:** 1 Health center- II, Isfahan University of Medical Sciences, Isfahan, Iran; 2 Department of Nursing and Midwifery, Isfahan (Khorasgan) Branch, Islamic Azad University, Isfahan, Iran

**Keywords:** breast cancer, sexual identity, sexual role, sexual function, sexual behavior

## Abstract

**Background:**

Despite the most of treatment team efforts focused on the maintaining patient’s life, attention to sexual issues don’t be considered. This stud is designed to determine the sexual behavior pattern and related factors in women with breast cancer.

**Methods:**

This descriptive- correlation study was performed on 90 women that diagnosed with breast Cancer that was admitted to Sayed-Al-Shohada hospital of Isfahan in 2010. Sampling method was available (non- random sampling) and Sexual Behavior Pattern determined with 3 domains: sexual identity, sexual role and sexual function. Data collection tools, was a questionnaire that made by the researcher and was used after determining the validity and reliability. For data analysis, was used of Descriptive- analytic statistics, frequency and ANOVA and Pearson correlation analytical tests in the SPSS statistical software (version 16).

**Results:**

Cases had 60% of Desirable sexual identity, 50% of Desirable sexual role, 40% Desirable sexual function and were be able to play 47.61% Desirable sexual behavior. Participants that their husbands had Elementary education had more desirable sexual behavior (p<0.031). Cases that were homemaker had more desirable Sexual behavior than of were working and retired (p<0.023). Non-surgical treatment with chemotherapy, radiotherapy and hormone therapy had a negative impact on sexual behavior (p<0.014).

**Conclusion:**

Study of sexual behavior pattern that is one of the important aspects of health, Provide valuable information to nurses and medical team and will be enhance the quality of provided services. Adopt appropriate strategies and interventions to promote sexual health, breast cancer is recommended.

## 1. Introduction

Breast cancer is the malignant proliferation of epithelial cells of the inner breast ducts (Harrison & Randlvf, 2007). In Iran and many countries, breast cancer is the most common cancer among women. Approximately, one million patients are diagnosed with breast cancer in the world (Hazrati, Poor Kiani, Abas Zadeh, & Jafari, 2008). With 6156 breast cancer in Iran, the provincial distribution is relatively uniform and is placed on the top of the cancer cases reported. 1 out of every 8 woman is having the chance of developing breast cancer (Aghajani, Mesdaghinia, & Emami, 2012). It is extensively known that due to particularly complex changes caused by cancer, sexual activity is often one fully problematic aspect of a woman’s life (Anders, Johnson, Litton, Phillips, & Bleyer, 2009). Investigating the situation of sexual behavior is an aspect of nursing cares which in the last decades, has been upgraded with developing the concept of comprehensive nursing (Lavin & Hyde, 2006) Sexual needs are not only a physiological need but are considered in the area of spiritual and mystical requirements and the need for beauty and perfection. Although the concept of sex international and global issue, it has by no means a unique interpretation and the expression is not absolute (Ohadi, 2002). Sexual behavior is an aspect of nursing care that has improved in recent decades, with the development of a global comprehensive nursing (Ohadi, 2002). To achieve more accurate recognition from the situation of sexual behavior of each individual, the person should be under investigation and evaluation in three main areas related to sexual behavior. These main areas are: 1- the person’s feeling about his gender identity. 2- The sexual role that the person plays in relationship with his sex partner. 3- The situation of sexual function (Kajbaf, 2003). Sexual behavior is the description the behavior of a person’s sexual identity, including the inclination and sexual relations between individuals. Generally, the important concepts in the sexual behavior include sexual identity, function and sexual role (Kajbaf, 2003). Sexual identity is formed on the basis of biology and social experiences and implicitly refers to psychological aspect of masculinity or femininity feelings. There is often a harmony between the concept of sexuality and sexual identity. Although, sometimes it also might have some digressions (Kajbaf, 2003). The sexual aspect of a person’s behavior is the reflection of the individual’s own feelings about her sexual identity. In other words, it is the role that the individual plays in terms of sexual characteristics in front of her husband (Jahanfar & Molaei-Nejad, 2002).

Sexual function describes the behavior of a person’s sexual identity and includes sexual relations between individuals. Many aspects of sexual function are effective on the patient’s health condition and are important in relation to nursing care and its results effects on the patient. Shepard and Ely (2008) study showed that sexuality is a profound aspect, comprehensive and coherent part of one’s personality and is the core basis of one’s sense of health and attitude towards oneself (Sheppard & Ely, 2008). The study of Ismaelzade and his colleagues (2009) showed that with due attention to the sexual desires of their husbands, women can improve the relationships between spouses and reduce stress in their family and vice versa, the lack of attention will lead to the following problems (Ismaeil-Zadeh, Qureishi, & Nazer, 2010). Discovering and solving sexual problems and helping to improve the quality of marriage, would have a great impact on increasing sexual satisfaction and prevention of family conflicts and the consequences resulting from the issue (Bolurian & Ganjloo, 2007).

Based on conducted investigations in Iran, a study has not been done on the status of sexual behavior and especially investigating the situation of the feeling of gender identity and sexual role of women after developing breast cancer. With consideration of the importance and impact of sexual issues on protecting the wives relations and also Due to the relatively long-term survival of women after breast cancer and their essential place at the preservation and survival of the family and the impact of sexual issues on relations between spouses, the research has had the goal to identify and obtain more information on sexual behavior pattern and its related factors in women with breast cancer.

## 2. Method of Survey

This research is a descriptive-analytical study. The research environment included the clinics, clinical departments, chemotherapy and radiotherapy of Imam Husain (PBU) Medical Center in Isfahan.

The subjects of study consisted of 90 patients of married women with breast cancer whose disease was at the 1-3 phases. These patients if referred to the center and, if married, with a peremptory cancer diagnosis by a doctor and registered diagnosis in the patient’s file saying that she is at 1 or 2 or 3 breast cancer in at least 3 months prior to the study and the lack of any history of psychiatric or other systemic chronic diseases that would cause any hindrance in the interviews process or the spread of cancer to any organs or recurrence of breast cancer, if they were interested they were being recruited in the course of the study.

Sampling was conducted with the method of accessibility and with the time interval in April to July in 2010. Data collection instrument was the researcher made questionnaire which consisted of 33 questions in 3 parts developed through surveying valid literature and articles in this field and was approved by scientific experts of the board members in this field. The first part of the questionnaire included demographic information, the second part consisted of the disease information of the case based on her profile and the third was related to sexual behavior in subject of the study. Questions related to sexual behavior pattern, included concepts of sexual identity, sexual role, and practices.

The design of the questions related to sexual function was based on ALARM Model. ALARM Model is one of the models which focuses on assessment of sexual function and has been developed by Anderson and Lamb (Krebs, 2008). This model investigates the type and level of sexual activity, libido and sexual desire and arousal, quality and amount of lubrication and the ability to achieve vaginal orgasm like receiving reciprocal peak of the sexual resolution. In addition, this model of disease and the existing disability level, the types of medication and side effects associated with treatment, and the common treatment of diseases and post-treatment have been studied (Anderson & Lamb, 2007). The questions were designed based on Likert Table with 5 scale measures including small allotment area, low, medium, high and very high which were rated from one to five. The number one includes the lowest score, and number five is receives the highest rating given to the question from the perspective of patients.

To determine the validity or reliability of the questionnaire, the content validity method was used. To obtain the validity or reliability of the questionnaire the Cronbach’s alpha test which is a method of approving the internal coherence was used. In this way, initially, pilot test was carried out and the numbers of 15 questionnaires were completed through the questioner’s interview with the patient. Data were entered into the statistical software of spss16 and its alpha coefficient was measured. The Cronbach’s alpha coefficient represented the scientific validity of the research device which was approved to be 94%. Data collection method was conducted through the interviews under the field category at the Medical Center of sayed-al- shohada and the interviews with women with breast cancer was conducted by the researcher himself.

Descriptive statistics determining the mean and standard deviation for data analysis were used for quantity analysis and frequency distribution table was used for the analysis of qualitative data and in order to examine the relationship between demographic and disease characteristics and the components of sexual behavior of the subjects under study, the analytical statistics and ANOVA data tests were used for comparison of more than 2 groups and Pearson’s correlation coefficient was used to assess the quantitative data in SPSS software version 16. P value <0.05 was considered significant.

The gained scores in both desirable and undesirable situation were calculated. Scores lower than 50% of the total score of the questionnaire means the undesirable situation and scored higher than 50% of the total score of the questionnaire was considered a favorable situation.

To comply with ethical considerations, with obtaining necessary agreements and adequate explanations after the informed consent of the subjects under study, the research was conducted.

## 3. Results

The findings of this research, in the field of individual specifications of units under study, showed that the average age of studied women was 48.24±6.87 and the average age of their husbands was 53.74±8.81.

The information about the individual specifications of units under study and their spouses is given in Table 1.

**Table 1:** The frequency distribution of individual specifications of units under study and their spouses

Type of job	The number of patients (percent)	The number of spouse (percent)
Housewife	72(80)	0.0
Employee	11(12.2)	13(14.4)
Retired	7(7.8)	25(27.8)
Self employed	0.0	34(37.8)
Laborer	0.0	17(18.9)
Unemployed	0.0	1(1.1)
Total	90(100)	90(100)

**Level of Education**	**The number of patients (percent)**	**The number of spouse (percent)**

Uneducated	26(28.9)	16(17.8)
Elementary school	33(36.7)	42(46.7)
Middle and high school	24(26.7)	22(24.4)
Highly educated	7(7.8)	10(11.1)
Total	90(100)	90(100)

According to the findings of Table (1), the maximum number of units under study (80%) was housewives and the lowest number of them (7.8%) was retired. Also, the highest number of husbands of units under study (37.8%) was self-employed. The largest number (36.7%) of units under study had primary levels of education and the lowest number of them (7.8%) was highly educated. Also, the highest number of husbands of units under study (46.7%) had primary levels of education and the lowest number of their husbands had higher education levels (11.1%).

According to the findings of Table (2), the maximum number of units under study (56.7%), were in the second stage of disease and the lowest number of them (21.1 %) were in the third stage of disease. The method for diagnosing disease in most numbers of studied units (85.6%) was done by the person and in the lowest number (2.2%), was performed by the medical team. Surgical treatment of radical mastectomy was done in the highest number (51.1%) of units under study and surgical treatment of lumpectomy was performed in the lowest number (25.6%) of them. In the greatest number of units under study (35.6%), non-surgical treatment, chemotherapy along with radiotherapy was done and in the minimum number of them (7.6%), only the non-surgical treatment of radiotherapy was performed.

**Table 2:** The frequency distribution of characteristics of disease in units under study

Disease stage	Number	Percent
Stage 1	20	22.2
Stage 2	51	56.7
Stage 3	19	21.1
Stage 4	0	0
Total	90	100

Method for diagnosing disease	**Number**	**Percent**

Self-examination	77	85.6
Examination by medical team	2	2.2
Methods of imaging	5	5.5
Biopsy	6	6.7
Total	90	100

The type of surgical treatment	**Number**	**Percent**

Lumpectomy	23	25.6
Total mastectomy	21	33.3
Radical mastectomy	46	51.1
Total	90	100

Non-surgical type of treatment	**Number**	**Percent**

Chemotherapy	21	23.3
Radiotherapy	6	6.7
Chemotherapy plus radiotherapy	32	35.6
Chemotherapy plus radiotherapy and hormone therapy	31	34.4
Total	90	100

The type of disorder	**Number**	**Percent**

Cessation of menstruation	55	66.1
Increase in bleeding	8	8.9
Bleeding between periods	1	1.1
Spotting	2	2.2
Irregular menstruation	4	4.4
No menstrual dysfunction	4	4.4
The menopause before the disease	16	17.8
Total	90	100

Investigating the situation of sexual behavior of units under study showed that they were able to perform 47.61% favorable sexual behavior.

Based on results obtained from Table (3), the units under study had 60% desired gender identity, 50% desired sexual role and 40% favorable sexual function and in total, they were able to perform 47.61% desirable sexual behavior.

**Table 3:** The mean and standard deviation of the Status of sexual behavior indicators in units under study

Statistical indicators	Sexual identity	Sexual role	Sexual function	Sexual behavior
Average score (percent)	15(60%)	15(50%)	20(40%)	50(47.61%)
Standard deviation	2.54	4.58	3.03	7.3

The results obtained from comparing the individual specifications of patients and their spouses and the sexual behavior of units under study showed that there is an inverse relationship between educational level of the patient’s husband and the patient’s sexual behavior. So that

Sexual behavior of the subjects whose husbands had elementary education was more favorable than the subjects whose husband were at guidance school or secondary education level (p<0.023).

## 4. Discussion

The research showed that units under study did not have a desirable sexual behavior, so that, they were able to perform 47.61% favorable sexual behavior. This finding is also compatible with other studies conducted in this regard (Fogel & Lauver, 1990; Anllo, 2000; Mock, 1993; Walsh, Manuel, & Avis, 2005; Lee, 2002; Traun-vogt & Herdina, 2010; Sheppard & Ely, 2008). In explaining this finding, it can be stated that breast cancer and its treatments have an undesirable impact on women’s mental picture of their body, which this issue could have a negative impact on their sexual relations. In the crisis experience caused by breast cancer, there are concerns about the treatment, surgery, chronic illness and definitive death and all these cases can make sexual activities to be relegated to the background. All attempts of spouses are used for compliance with medical problems. Therefore thoughts about sexual issues seem inappropriate and there is a desire to discuss these issues in the future. On the other hand, sexual activities can have different meanings for different people and even can change for a person from time to time.

In this study, there was an inverse relationship between the wife’s educational level and desirable sexual behavior of units under study. The sexual behavior for units under study that their husbands had lower levels of education was more desirable than units with husbands who had higher education levels. In explaining this finding, it can be stated that due to this fact that most of the units under study in this research (36.7%) had primary levels of education, probably, they don’t have much knowledge about the type of the disease and the complications that could arise in the future and therefore, the current situation will be more tolerable for them than other units under study and this lower levels of stress and anxiety had a lower impact on their sexual behavior (Rashidi, 2010). These findings is an inconsistency with the results of Manganilo et al. (2010) study showed that there is a positive relationship between sexual performance and increasing years of education (Manganiello, Hoga, Roberte, Miranda, & Rocha, 2010).

These mentioned findings can be explained as follows that the husbands with primary education are less likely to be able to understand the conditions created for their wives and while women need to receive emotional support and sympathy from his wife, these partners continue insisting to receive their natural right that the law has set for them and women have been forced to comply and follow their husbands. On the other hand, given that most of the subjects in this study (36.7 percent) have primary education, probably, have little knowledge of the disease and the complications that could arise in the future for them and therefore they are more tolerable to the conditions in compare to other subjects and they have lower levels of stress and anxiety and less likely to influence their sexual behavior (Abedi, Rostami, & Nikpor, 2012). In this study, subjects under study were housewives whose sexual behavior was more desirable than the subjects who are retired.

Nazary (2007) quotes Hall study (1996) and states that with the massive increase in the role of working women, sexual dissatisfaction also increases. The life of couples who are both working, causes a lot of stress and is full of energy and the pressures of life caused them to withdraw from each other. Most of these spouses do not have the social skills, friends, school, working place, religious institutions and schools also do not support families in which both people work (Nazari, 2008).

Sexual behavior of the participants who had non-surgical radiation treatment, are more desirable than the patients who had non-surgical treatment of chemotherapy along with radiation and hormone therapy.

The findings of this study are compatible with Continelli et al. (2006) and Young (1996) and Heravi Karimi et al. (2004) that showed the results of their study indicates non-surgical treatment of breast cancer has adverse effects on sexual behavior of women with breast cancer (Cantinelli et al., 2006; Young & Caughan, 1996; Heravi-Karimvy, Jadid Milani, Forotan, & Aein, 2007).

In explaining the findings from this study, it can be expressed that the non-surgical treatments such as chemotherapy and hormone therapy can have adverse effects on ovarian function. Menopause, nausea and vomiting, severe change in appearance and general health of the patient, cause long-term effects on the sexual behavior of women with breast cancer.

## 5. Conclusion

International Conference on Population and Development in Cairo (1999) considers the right of all people to achieve the highest standard of sexual health information and sexual activity and proper training are essential components of standard health care (Anders, Johnson, Litton, Phillips, & Bleyer, 2009). Women are the most important element of the family and community. Thus, according to the needs and physical and psychological aspects of women with breast cancer, they will not only improve their survival but also increases the quality of life and causes the greater cohesion in the family structure (Aghabarari, Ahmadi, Mihammadi, Haji-Zade, & Varvani-Farahani, 2007). For sexual health there should be a balance between genetic gender, glandular sexuality, genital organ, physical appearance and psychosexual characteristics (gender identity, sexual tendency and sexual role) (Kula & Slowikowska, 2000).

Providing a model of sex reconstruction after breast cancer and conveying development of psycho-dynamic mental model creates an opportunity to advance in the treatment of cancer. The model shows when and how those conflicts increase and how these conflicts will match (Traun-Vogt & Herdina, 2010).

Considering the fact that the nurses spend more time with patients than other health care team members, they have an effective role in promoting health and sex conditions of the patients and nurses of cancer wards must be more aware of the impact of breast cancer on sexuality and when discussing these issues with patients and their spouses should be more at easy to help patients to fix their problems.

**Limitations of the Research**

Investigating the sexual behavior, outside the form of marriage is faced with lots of limitations. Therefore, this study examines the sexual behavior only in relation to official spouse. As a result, checking individuals in terms of homosexuality feelings and having relationships outside of marriage is not possible and this study, in this regard is faced with lots of limitations. Other studies in this area are recommended.
